# Comparative Evaluation of the Quality of Obturation Between Three Obturation Techniques in Primary Canines: An In Vitro Study

**DOI:** 10.7759/cureus.63845

**Published:** 2024-07-04

**Authors:** Manal Almutairi, Reema Almutairi, Shaikha Almogbel, Atheer Alfarhan, Wajd Alharbi, Rawan A Alayoub

**Affiliations:** 1 Pediatric Dentistry, King Saud University, Riyadh, SAU; 2 College of Dentistry, King Saud University, Riyadh, SAU; 3 General Dentistry, King Saud University, Riyadh, SAU

**Keywords:** primary teeth, zinc oxide-eugenol cement, root canal obturation, pulpectomy, metapex, digital radiography

## Abstract

Aims

This study compares three obturation techniques (rotary lentulo spiral, handheld lentulo spiral, and pressure syringe) for the quality of two filling pastes (zinc oxide eugenol (ZOE) paste and Metapex (Meta Biomed Co., Ltd., Chungcheongbuk-do, Korea).

Methods and materials

Sixty extracted primary canines were instrumented and obturated by filling materials. The obturation techniques were divided into three groups according to different obturation techniques. Obturation quality was evaluated for length, density, and presence of voids by using digital radiography.

Results

This study showed that the handheld lentulo spiral technique using Metapex and ZOE exhibited more optimal fillings for obturation length. The highest density obturation was achieved using the syringe injection approach with Metapex and ZOE. The highest incidence of both external and internal voids was observed in the group using ZOE with the handheld lentulo spiral technique

Conclusions

Based on the findings of this study, for both filling materials, the handheld lentulo spiral technique had the greatest number of optimal lengths but there were also more voids.

## Introduction

Preserving the primary dentition is crucial to maintaining space for permanent tooth eruption [1]. A pulpectomy is one option for treating primary teeth with radicular pulpal tissue inflammation or loss of vitality until their regular exfoliation period [1,2]. It involves canal obturation with resorbable antibacterial paste after removing infected pulp tissue [2]. Aseptic root canal preparation followed by a hermetic seal in obturation is essential for successful pulpectomy treatment in primary teeth with necrotic pulps [3,4].

Root canal obturation in primary teeth differs from that in corresponding permanent teeth. One of the essential requirements of the root canal filling material is that it should resorb at the same rate as the primary tooth roots [5]. The ideal obturation procedure ensures that the root canal is filled without overfilling, with few or no voids; the filling material should be non-toxic to the permanent tooth germ or periapical area in the case of overfilling accidents [6,7]. Some examples of obturation materials for primary teeth are zinc oxide eugenol (ZOE), calcium hydroxide, and iodoform pastes. Despite its widespread use, zinc oxide eugenol's toxicity and slow resorption rate raise significant concerns regarding its safety and efficacy. Therefore, alternative obturating materials, such as calcium hydroxide and iodoform combinations, have been developed to overcome these problems [7,8].

Calcium hydroxide demonstrates antiseptic properties, osteoconductive potential, and a rapid resorption rate (a drawback because the resorption rate is faster than the physiological root resorption of primary teeth) [7]. Metapex (Meta Biomed Co., Ltd., Chungcheongbuk-do, Korea), composed of calcium hydroxide and iodoform, has antibacterial properties and is preferred by clinicians for ease of use [9,10]. These obturation materials can be delivered by pluggers, disposable injections, handheld or rotary lentulo spiral, and endodontic pressure syringes [11].

It is challenging to obtain a void-free root canal filling in clinical pulpectomy practice today, and the success of pulpectomy in primary teeth depends on obturation quality. Therefore, there is no consensus in the literature about the approach offering the best seal for the root canal system in primary teeth [12-14]. This study aimed to compare the quality of three obturation techniques (rotary lentulo spiral, handheld lentulo spiral, and pressure syringe) for two fillings (ZOE paste and Metapex) in primary canines using digital radiography to evaluate the quality of obturation.

The null hypothesis is that there is no difference in the quality of obturation of zinc oxide eugenol and Metapex when evaluated with digital radiography.

## Materials and methods

Under research number E-22-6862, the Ethics Committee of King Saud University, Riyadh, Saudi Arabia, accepted the study protocol of the College of Dentistry Research Center. For this in vitro investigation, the determined sample size was at least 60 teeth/10 in each group at the significance level of σ = 0.05 with an e size of 0.5 through power= 0.95.

The study included 60 extracted primary canines. Following radiography, teeth were included if they had at least two-thirds remaining root, were free of internal resorption or calcification, and showed no dental abnormalities. Following extraction, the teeth were rinsed with water, placed in 2.5% sodium hypochlorite for a week, and then immersed in distilled water for storage [15].

A 1012 diamond spherical drill (KG Sorensen, Cotia, Sao Paulo, Brazil) was used to access the palatal surface for all extracted primary canines. A size #15 Kerr file (Mani Co., Tokyo, Japan) was used for determining the working length, which was then adjusted to 1 mm short of the actual length by visualizing the root canal apex. The ProFileR (Dentsply, Maillefer, Ballaigues, Switzerland) reciprocating instrument was used in three pecking movements to clean and shape the root canal, followed by irrigation until the working length was reached. The instrument was gradually increased to 30-35/0.04 size using a rotary handpiece (Bien-Air Dental, Langgasse 60, Biel, Switzerland) at 300 rpm with minimum torque. Following each filing procedure, 10 milliliters of normal saline was used for root canal irrigation. Subsequently, the canals were dried using absorbent paper points (Spident, Incheon, Korea). Only five root canals were prepared per session to minimize operator fatigue-related biases. One trained operator performed all the root canal preparations.

The prepared root apices were covered with remodeling wax (Golden Modelling Wax, Telangana, India) to create a halo zone, which would function as a gathering area for any extruded canal-filling material. The root ends were set in 2 × 2 cold-cured acrylic resin blocks (Major Ortho, Major Prodotti Dentari S.p.A., Moncalieri, Italy) beginning 1 mm below the cementoenamel junction.

Obturation technique

Before initiating obturation, the canals were irrigated and dried using paper points. The filling pastes, ZOE paste (Pulpdent, Watertown, MA, USA) or MetapexR (Meta Biomed Co.), and the application techniques were used to randomly split the 60 teeth into 6 groups (n=10) as follows:

Group 1: Prepared canals that were injected by syringe with MetapexR. First, the disposable tip was inserted in the canal before it was pressed for use. The syringe was slowly withdrawn and pushed again until the paste filled the canal orifice after reaching the apex.

Group 2: Prepared canals that were filled with MetapexR using a handheld lentulo spiral (Dentsply, Tulsa, OK, USA) rotated clockwise into the canal, pushed out of the canal using vibrations to allow the material to reach the apex, and then put back into the canal. This operation was repeated until the dispensed cement filled the canal orifice.

Group 3: MetapexR was inserted into the prepared canals using a motor-driven lentulo spiral (Dentsply, Tulsa, OK, USA). Following working length, a lentulo spiral size #30 was mounted on a slow-speed handpiece, coated with Metapex R, placed into the canal, and slowly retreated while continuing rotations (3-5 repeats of this process) until the canal orifices were filled.

Group 4: ZOE was injected into prepared canals by mixing one volume unit of powder and two volume units of liquid on a dry glass slab at room temperature. The mixture was mixed for 45 seconds to create a creamy consistency. In the same technique as group 1, the ZOE paste was put into a 3 ml syringe (Crystal-Ject, Bu Kwang Medical Inc., Korea) and connected with a disposable tip (Meta Biomed Co.) to fill the canal.

Group 5: ZOE was filled to prepare canals using a manual lentulo spiral (Dentsply, Tulsa, OK, USA) technique, similar to group 2.

Group 6: ZOE was filled into prepared canals using a motor-driven lentulo spiral (Dentsply, Tulsa, OK, USA) technique, similar to group 3.

A rubber stopper was positioned around each instrument at a distance decided by preoperative measurements to control paste delivery in each group. A moist cotton pellet was used to gently seal the material into the canals until all group canals were filled. Subsequently, a thick ZOE mixture was inserted into the access cavity.

Radiographic evaluation

Following pulpectomy, teeth were evaluated using digital radiography at standardized kilovolts (70 kVp) and 6 mA milliamperes for an exposure time of 0.160 s with a target-film distance of 15 mm to minimize radiographic errors. A radiographic examination was performed using a paralleling cone technique in buccolingual and mesiodistal orientations, with each tooth in all six groups having a similar alignment of its receptor and tooth. This method allowed for accurate and consistent radiographs that enabled optimal tooth structure visualization and reproducibility. A skilled pediatric dentist and a dental intern blinded to the filling process independently assessed the radiographic quality of obturation for length and density using modified methods of Coll and Sadrian (1996), Sigurdsson et al. (1992), and Somma et al. (2011) [16-18].

The measurement of the obturation length in filled root canals was done by considering the distance between the filling and the apex [16]:

Underfilling: The canal filled more than 2 mm short of the apex.

Optimal filling: Canal with filled ending at radiographic apex up to 2 mm short of the apex.

Overfilling: If a canal is filled beyond the root apex.

**Figure 1:**
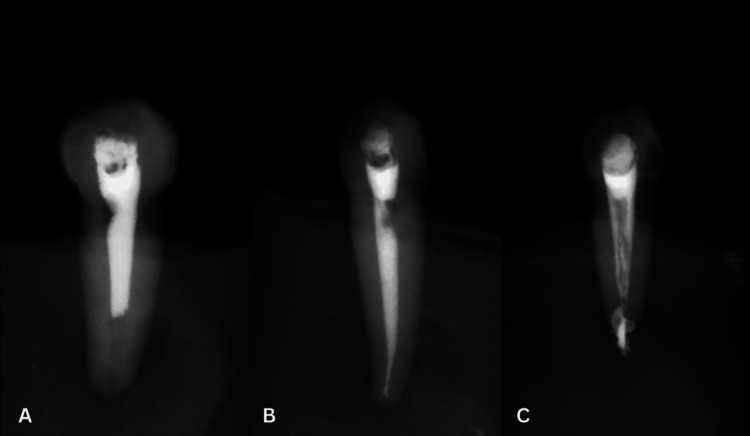
Radiographs showing the obturation's length A. Underfilling. B. Optimal filling. C. Overfilling

Figure 1 shows the obturation length based on the above factors.

The density of the root canal filling was determined as follows [17]:

Unacceptable: If there are more than three voids at different root canal locations.

Acceptable: Successful filling, with one to three voids observable.

Good: Perfect filling, with no voids.

**Figure 2:**
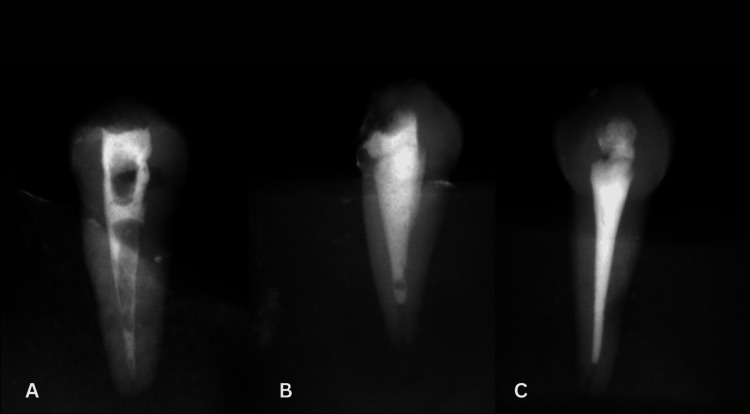
Radiographs showing the obturation density A.Unacceptable. B. Acceptable. C. Good

Figure 2 displays the radiographs showing the obturation density based on the above factors.

The following factors were used to determine the distribution presence of voids [18]:

Internal voids: Distributed void inside the filling material.

External voids: Distributed voids along the canal walls.

Internal and external voids: Voids present with internal and external canal walls.

No voids: Absence of voids.

**Figure 3:**
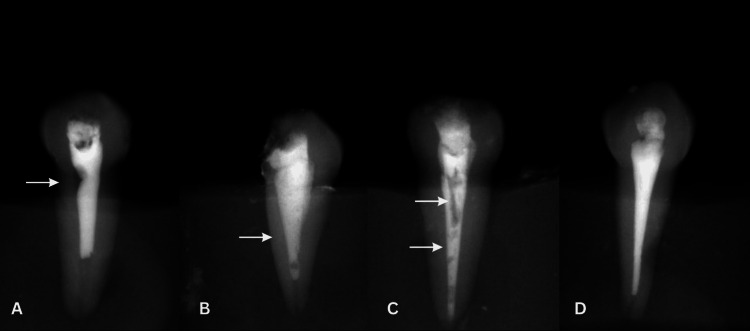
Classification of voids A. External void. B. Internal void. C. Internal and external voids. D. No voids.

Figure 3 displays the classification of voids.

Statistical analysis

The chi-square test was used for statistical analysis of the data. When the chi-square test showed significant results, the Kruskal-Wallis H test was used to compare the obturation quality between the different procedures. Version 22 of the Statistical Package for Social Sciences (SPSS; IBM Corp., Armonk, NY, US) was used for data analysis. A significant level of p < 0.05 has been established.

## Results

Utilizing Cohen's kappa statistic, the inter-rater reliability of the void classification and quality of obturation was assessed. The results revealed an outstanding agreement between the two observers of (95% CI: 0.91-1), p = 0.00.

The obturation quality was evaluated for length and density. Table 1 shows the frequency distribution of all groups categorized by these variables. For obturation length, there was no statistically significant difference between all groups (p>0.05). The highest number of teeth with optimal fillings was found in Group 5 (ZOE by handheld lentulo spiral, 60%), followed by Groups 1 and 2 (Metapex by injection technique and handheld lentulo spiral, 50%) and Group 3 (Metapex by motor-driven lentulo spiral, 40%). In contrast, there was a significant difference in the density of all groups (p<0.05). Groups 1-4 (Metapex by injection technique, handheld and motor-driven lentulo spiral, and ZOE by injection technique) had the highest number of teeth with an acceptable density of obturation (70%), followed by Group 5 (ZOE by handheld lentulo spiral, 50%). However, Group 6 (ZOE by motor-driven lentulo spiral) had 60% of teeth with an unacceptable density of obturation.

**Table 1:** Distribution of all groups according to the quality of obturation p < 0.05 is statistically significant.

Groups of obturation	Quality of obturation											
	Length of obturation							Density of obturation				
	Under-filling	Optimal filling	Over-filling	Total		P value	Unacceptable		Acceptable	Good	Total	P value
Group 1: Metapex by injection technique.	4(40.0)	5(50.0)	1(10.0)	10(100.0)	0.757			1(10.0)	7(70.0)	2(20.0)	10(100.0)	0.027*
Group 2: Metapex by handheld lentulo spiral.	4(40.0)	5(50.0)	1(10.0)	10(100.0)				3(30.0)	7(70.0)	0(0.0)	10(100.0)	
Group 3: Metapex by motor-driven lentulo spiral.	4(40.0)	4(40.0)	2(20.0)	10(100.0)				3(30.0)	7(70.0)	0(0.0)	10(100.0)	
Group 4: ZOE by injection technique.	4(40.0)	2(20.0)	4(40.0)	10(100.0)				3(30.0)	7(70.0)	0(0.0)	10(100.0)	
Group 5: ZOE by handheld lentulo spiral.	2(20.0)	6(60.0)	2(20.0)	10(100.0)				5(50.0)	5(50.0)	0(0.0)	10(100.0)	
Group 6: ZOE by motor-driven lentulo spiral.	4(40.0)	3(30.0)	3(30.0)	10(100.0)				6(60.0)	1(10.0)	3(30.0)	10(100.0)	

The presence of voids in all groups is shown in Table 2. Most teeth in all groups had voids. The highest number of teeth with external and internal voids was in Group 5 (ZOE by handheld lentulo spiral, 70%), followed by Group 6 (ZOE by motor-driven lentulo spiral, 60%) and Group 2 (Metapex by handheld lentulo spiral, 50%). There was a statistically significant difference across all groups regarding the presence of voids (p>0.05).

**Table 2:** Distribution of all groups according to the presence of voids p < 0.05 is statistically significant.

Groups of obturation	Presence of voids					P value
	External voids	Internal voids		External and internal voids	No voids	
Group 1: Metapex by injection technique	4(40.0)		0(0.0)	3(30.0)	3(30.0)	0.001
Group 2: Metapex by handheld lentulo spiral	4(40.0)		10(10.0)	5(50.0)	0(0.0)	
Group 3: Metapex by motor-driven lentulo spiral	1(10.0)		6(60.0)	3(30.0)	0(0.0)	
Group 4: ZOE by injection technique	0(0.0)		6(60.0)	4(40.0)	0(0.0)	
Group 5: ZOE by handheld lentulo spiral	1(10.0)		2(20.0)	7(70.0)	0(0.0)	
Group6: ZOE by motor-driven lentulo spiral	0(0.0)		1(10.0)	6(60.0)	3(30.0)	

Table 3 showed that data analysis using the Kruskal-Wallis test revealed no significant differences between all groups for the length, density, and presence of voids during obturation.

**Table 3:** Mean and sum rank scores in all groups p < 0.05 is statistically significant.

Quality of Obturation	Groups of Obturation	Mean Rank	Kruskal-Wallis H	P value
Length of obturation	Group 1: Metapex by injection technique.	28	1±514	0±911
	Group 2: Metapex by handheld lentulo spiral.	27±50		
					Group 3: Metapex by motor-driven lentulo spiral.	29±40
	Group 4: ZOE by injection technique.	33±20				
					Group 5: ZOE by handheld lentulo spiral.	34±10
	Group 6: ZOE by motor-driven lentulo spiral.	31±30				
					Density of obturation	Group 1: Metapex by injection technique.	39±65	5±247	0±386
	Group 2: Metapex by handheld lentulo spiral.	30±25							
						Group 3: Metapex by motor-driven lentulo spiral.	30±25		
	Group 4: ZOE by injection technique.	30±25							
						Group 5: ZOE by handheld lentulo spiral.	24±75		
Group 6: ZOE by motor-driven lentulo spiral.	27±85								
		Presence of voids	Group 1: Metapex by injection technique.	31±60		9±963	0±076		
Group 2: Metapex by handheld lentulo spiral.	24±30								
			Group 3: Metapex by motor-driven lentulo spiral.	23±80					
Group 4: ZOE by injection technique.	27±30								
			Group 5: ZOE by handheld lentulo spiral.	32±60					
Group 6: ZOE by motor-driven lentulo spiral.	43±40								

## Discussion

Pulpectomy is an endodontic procedure for treating primary teeth with irreversible pulpitis [19]. One of the main objectives of pulpectomy is to obtain an optimal filling that fills the entire root canal system without any voids or gaps [16]. Since there was a significant difference between ZOE and Metapex according to obturation techniques, the null-all in vitro hypothesis was partially rejected (p < 0.05).

Thus, pulpectomy maintains the functionality of the primary tooth, eliminates the source of infection and inflammation, and relieves the pain caused by pulpal tissue injury. The tooth continues to function until the eruption of the permanent successor [20].

Numerous studies have evaluated the effectiveness of various root canal filling techniques in primary teeth; however, there are varying opinions on the method that provides the best seal for the root canal system of primary teeth [7-10]. Therefore, this study compared the quality of three obturation techniques (rotary lentulo spiral, handheld lentulo spiral, and pressure syringe) for the quality of two fillings (ZOE paste and Metapex) in primary canines using digital radiography to evaluate the quality of obturation.

Both conventional and recent digital radiography were used to evaluate the quality of root canal treatment and the outcome [21,22]. Digital radiography is preferable to traditional radiography due to its minimum radiation exposure to the patient, the ability to gain access to images immediately, the elimination of the need for photographic processing, and the image modifications are unnecessary such as magnification digital imaging modalities have been proven to be more efficient than analog and CBCT in locating the presence of smaller voids [23,24].

In the present study, the results of the length of obturation indicated that using ZOE inserted by handheld lentulo spiral yielded the best results in achieving optimal length, which is consistent with previous studies by Memarpour et al. [22] and Vashista et al. [25], which demonstrated that the handheld lentulo spiral technique performed better in terms of optimally filled root canals minimizing material extrusion. Also, similar results were seen by Dandashi et al. [26], who compared lentulo spiral, pressure syringe, and incremental techniques. They observed that the lentulo spiral and incremental techniques exhibited less material extrusion. However, Oztan et al. found that teeth obturated with lentulo spirals were considered underfilled while other canals obturated with the previous inject technique achieved optimal canal filling length [27].

The optimal density of root canal filling is crucial to ensure a long-lasting therapeutic option. Inadequate density and inhomogeneous fillings may result in treatment failure [28]. Our study found that the densities of obturation in two groups were unacceptable: the teeth with ZOE inserted by motor-driven lentulo spiral and those with ZOE inserted by handled lentulo spiral had the worst densities. This finding is similar to a study by Asokan et al., which observed that root canal fillings performed with handheld lentulo spirals were less dense than those obtained via a syringe system [29]. This result differs from the studies by Nagaveni et al. [4] and Sigurdsson et al. [17], in which a lentulo spiral was considered the most effective for the obturation of primary teeth.

However, the density for all obturation techniques in our study using Metapex was considerably acceptable, in alignment with other studies by Subramaniam et al. [30], which reported a higher percentage of success with Metapex than ZOE and Endoflas. Previous studies also showed that Vitapex and Metapex provided better results than ZOE for root canal obturation [31,32].

The current study showed the presence of voids in the fully obturated canal in all groups. Factors that influence the presence of voids include the entrapment of air bubbles in the mixture during the mixing of the powder and liquid; additionally, a lentulo spiral may be repeatedly removed and reinserted throughout the root-filling process, or the operator may apply excessive pressure to fill all the gaps, which can result in voids [33,34]. However, the highest number of voids were seen in ZOE inserted by handheld lentulo spiral, followed by ZOE inserted by motor-driven lentulo spiral; whereas, in other groups, the differences were insignificant. Similarly, these findings are consistent with a prior study showing that the lentulo spiral group had the most voids and the insulin syringe group had the least [35]. Vasishta et al. [25] and Gandhi et al. [11] found more voids in the lentulo spiral technique, whereas Peters et al. found fewer voids in the lentulo spiral method compared to the injection system with Ca(OH)2 obturation [35]. The results of this study should consider the limitations, which include the use of only one type of tooth (canines), a small sample size, differences in operator experience, and a digital radiography method of evaluation that only provides a two-dimensional image of the canal space. The criteria for evaluation used in this study were another issue to take into consideration. We recommend that future studies encompass various types of teeth and canal configurations due to the variations in dental anatomy. Additionally, larger sample sizes, evaluation in three-dimensional images (Micro-CT), and in vivo studies should be conducted.

## Conclusions

The study's findings indicate that the handheld lentulo spiral technique, when utilized with Metapex and ZOE, yielded the most favorable results in terms of obturation length. In contrast, the syringe injection technique using the same materials produced the highest obturation density. Notably, the ZOE applied via a motor-driven lentulo spiral was associated with the highest percentage of unacceptable obturation densities. Despite all groups demonstrating the presence of voids, the application of ZOE through a handheld lentulo spiral was particularly prone to causing both external and internal voids.
